# Development of Fish Parasite Vaccines in the OMICs Era: Progress and Opportunities

**DOI:** 10.3390/vaccines9020179

**Published:** 2021-02-20

**Authors:** Saloni Shivam, Mansour El-Matbouli, Gokhlesh Kumar

**Affiliations:** 1 Clinical Division of Fish Medicine, University of Veterinary Medicine Vienna, 1210 Vienna, Austria; salonis@staff.vetmeduni.ac.at (S.S.); mansour.el-matbouli@vetmeduni.ac.at (M.E.-M.); 2 Central Marine Fisheries Research Institute, Karwar 581301, India

**Keywords:** fish parasites, immune response, omics, vaccines

## Abstract

Globally, parasites are increasingly being recognized as catastrophic agents in both aquaculture sector and in the wild aquatic habitats leading to an estimated annual loss between 1.05 billion and 9.58 billion USD. The currently available therapeutic and control measures are accompanied by many limitations. Hence, vaccines are recommended as the “only green and effective solution” to address these concerns and protect fish from pathogens. However, vaccine development warrants a better understanding of host–parasite interaction and parasite biology. Currently, only one commercial parasite vaccine is available against the ectoparasite sea lice. Additionally, only a few trials have reported potential vaccine candidates against endoparasites. Transcriptome, genome, and proteomic data at present are available only for a limited number of aquatic parasites. Omics-based interventions can be significant in the identification of suitable vaccine candidates, finally leading to the development of multivalent vaccines for significant protection against parasitic infections in fish. The present review highlights the progress in the immunobiology of pathogenic parasites and the prospects of vaccine development. Finally, an approach for developing a multivalent vaccine for parasitic diseases is presented. Data sources to prepare this review included Pubmed, google scholar, official reports, and websites.

## 1. Introduction

Aquaculture continues to be one of the rapid food-producing sectors worldwide. According to an estimate, the latest global aquaculture production was 82 million tons and valued at 250 billion USD in 2018 [1]. However, the sector is frequently hit by several viral, bacterial, and parasitic diseases with devastating consequences [2]. Although viruses and bacteria have been recognized as the leading cause of huge economic losses to the sector, the role of parasites has been realized recently. Growing literature suggests a considerable increase in parasitic epidemics both in farmed and wild fish populations. Parasites belonging to different groups such as myxozoa, protozoa, crustaceans, monogeneans, and helminths result in heavy losses in aquaculture and consequently to the allied industries. As per a report, the annual global loss of juvenile fish on account of parasitic infections was estimated to vary from 107.31 to 134.14 million USD and loss of marketable size fish from 945.00 million to 9.45 billion USD, the total estimate being 1.05 billion to 9.58 billion USD [3].

The management of parasitic infections in culture facilities involves different strategies such as quarantine, disease-free sites, disinfection of water using UV radiation and chemicals, fallowing, and drying of pond bottoms [4]. Instead of ponds, concrete tanks or raceways have been used for fish culture to prevent infection by myxozoan parasites in order to restrict the oligochaetes, which serve as the alternate hosts of these parasites [5]. Nevertheless, early efforts of controlling a parasitic infection in fish relied heavily on the use of chemotherapeutics. Consequently, their relentless use is leading to the emergence of drug resistance [6,7] and deleterious environmental effects [8,9]. Although phytotherapy-based treatment options are favorable, they suffer from various disadvantages [10,11,12,13]. Lately, the use of attractants and traps has been suggested as a promising strategy for certain parasites such as sea lice, by exploiting their chemotactic and phototactic responses [14]. Furthermore, a study highlighted the use of urea and light-based traps for controlling the infection by *Cryptocaryon irritans* and *Neobenedenia girellae* in aquaculture [15]. However, at present, application of this approach in commercial aquaculture is limited due to the unavailability of efficient traps. Vaccination is considered the best method for safeguarding and promoting fish health and welfare against any parasite. Although several commercial vaccines are available for bacterial and viral diseases globally [16], only Chile has a commercial parasite vaccine against sea lice [17]. The development of parasite vaccines is limited by several inherent issues. One of the most important factors is the biological complexity of the parasite. The parasite life advances through different developmental stages, which may have a specific antigen profile. Moreover, the life cycle stages alternate between different host species in various parasites [18], thus, interfering with the culturing and maintenance of parasites under laboratory conditions owing to requirements for optimization of several parameters such as temperature and nutritional elements for the alternate host, giving rise to more labor requirement and increased economic costs.

Omics studies are powerful methods for developing vaccines by providing potential vaccine candidates. The suffix “omics” refers to the high-throughput analysis of cellular macromolecules. The most popular omics disciplines include genomics, transcriptomics, and proteomics. The omics era started with genomics, aiming to study the entire gene content (genome) of an organism [19]. The genomic analysis provides abundant information on individual genes, chromosomes, their organization, genetic variants of diseases as well as evolutionary relationships with other phyla and parasites. However, genomics does not provide information on aspects such as gene expression, function and regulation, and structure and characteristics of encoded proteins [20]. These limitations have resulted in the advent of the post-genomic era primarily dominated by transcriptomics and proteomics. A transcriptome comprises all RNA transcripts produced by the genome under a given environmental condition [21]. Transcriptomic profiling provides information on different categories of transcribed RNAs, the transcriptional structure of genes, and the expression of genes [22]. Nonetheless, transcriptomics does not reflect the actual protein complement due to the many events in translation of mRNA transcripts, e.g., post-transcriptional modifications and alternate splicing [23]. Proteomics followed transcriptomics, which is defined as the study of proteomes (the entire set of proteins, that are the key players in biological processes) [21].

Genomic and transcriptomic analyses of certain fish parasites have been performed. In addition, transcriptomics-based studies have been conducted to investigate the host–parasite interaction in some instances [24]. Similar to human and veterinary parasitology, omics data could provide potential therapeutic targets against aquatic parasites.

In this review, we highlight the need and the progress made in developing parasitic vaccines, with particular focus on the immunobiology of fish parasites. Furthermore, we discuss the present status, the prospects of developing successful parasite vaccines in the present era of omics, and an approach for developing multivalent vaccines.

## 2. Data Sources, Searches and Study Selection

Searches were performed on pubmed, google scholar and google with the keywords either alone or in combination “parasite vaccines”, “fish parasites”, “transcriptomics”, “genomics”, and “proteomics”. According to the PRISMA (Preferred Reporting Items for Systematic Reviews and Meta-Analyses) guidelines, peer-reviewed articles were initially selected. The articles were then screened based on title and the abstract. Only full text articles were included in the study. Official reports of FAO (Food and Agriculture Organization of the United Nations), NCBI (National Center for Biotechnology Information) and OIE (Office International des Epizooties) websites were also referred to. Flow diagram is provided as Supplementary Data (Figure S1).

## 3. Economically Important Fish Parasites

The majority of the important fish endoparasites belong to the phylum Cnidaria, whereas the ectoparasites belong to phyla Ciliophora and Arthropoda (Table 1). The phylum Platyhelminthes encompasses both endoparasites and ectoparasites. Parasitic diseases in aquaculture cause both direct and indirect losses. Direct losses result from the mortality in farmed fish because of parasite outbreaks. Indirect losses are attributed to the investment made for treating infections, adopting management strategies, reduced growth resulting from infection, and the cost involved from carcass spoilage at harvest [3,18,25].

Some notable examples of commercially important myxozoan endoparasites include *Tetracapsuloides bryosalmonae, Myxobolus cerebralis, Sphaerospora molnari, Ceratomyxa shasta*, *Kudoa thyrsites*, and *Enteromyxum* sp. Proliferative kidney disease (PKD), caused by *T. bryosalmonae*, could result in up to 95% mortality in farmed salmonids [26]. *C. shasta* causes ceratomyxosis in chinook and coho salmon [38]. In addition, several other myxozoan parasites incur huge direct and indirect economic losses, e.g., *Myxobolus cerebralis* [5] and *Enteromyxum* sp. [39]. Some ectoparasites responsible for significant mortality and thus aquaculture losses include *Ichthyophthirius multifiliis*, *C. irritans*, *Argulus*, *Lepeophtheirus salmonis*, and *Caligus rogercresseyi*. Protozoan parasites *I. multifiliis* and *C. irritans* cause white spot disease in freshwater and marine fishes, respectively [18]. The crustacean parasites *L. salmonis* and *C. rogercresseyi,* commonly referred to as sea lice, cause severe infection in salmonids [40]. *Argulus* is the freshwater counterpart of sea lice reported from several fish hosts [41]. Furthermore, several parasites have been implicated in the decline of wild fish populations, e.g., *M. cerebralis* [42], *T. bryosalmonae* [43], *C. shasta* [44], *I. multifiliis* [45], *L. salmonis* and *C. rogercresseyi* [40].

Fish species of aquaculture importance differ with countries. Moreover, parasites causing mortality and incurring economic losses may differ. However, certain parasites are of global concern, e.g., *M. cerebralis, I. multifiliis*, and *Argulus* [18]. Despite reports of huge economic losses and fish mortality due to parasitic infections [18], their estimations are either lacking or are specific to locations. For instance, losses in salmonid farming accounted for 6 million Canadian dollars (CAD) in 2015 due to *K. thyrsites* infection in British Columbia, Canada [33]. *Argulus* is reported to cause losses amounting to 5.41 million United States dollars (USD) annually in carp aquaculture in India [31]. Globally, annual indirect and direct losses in salmonid aquaculture due to infestations with the sea louse *L. salmonis* have been estimated to be 500 million to 1 billion USD [46].

## 4. Progress in Understanding of Host-Parasite Interactions

Teleosts are the lowest vertebrates that elicit both innate and adaptive immune responses against pathogen invasion [47]. However, compared to higher vertebrates, innate immunity plays a major role than the adaptive immune response in aquatic organisms, irrespective of the nature of the pathogen [48]. Remarkably, innate immune reactions are not only the first line of defense but also set the pace for ensuing adaptive immune responses. Thus, the development of an effective vaccine requires a good understanding of immune response during host–parasite interaction and pathogenesis mechanisms. Some progress has been made in understanding the immune response in fish against parasitic diseases. Studies using immunoassays, gene expression, Western blotting, and other techniques have elucidated the expression of different molecules in innate and adaptive immune responses during several parasitic infections. The generation of an elevated immune response upon re-infection in fish, which could survive after previous natural parasite infection, has also been reported. Few examples include endoparasites, e.g., *T. bryosalmonae*, *C. shasta*, *E. scophthalmi*, *E. leei* [49] and ectoparasites, e.g., *I. multifiliis* and *C. irritans* [45,50]. Similar to other vertebrates, the development of immune memory in fish infected with parasites forms the fundamental basis of vaccine development.

### 4.1. Innate Immune Response during Selected Parasitic Infections

Studies on the immune response generated by parasites during infection and post-immunization, report the involvement of toll-like receptors (TLRs), phagocytes, complement proteins, melanomacrophage centers, proteases, and cytokines. TLRs are type-I transmembrane receptors that recognize pathogen-associated molecular patterns to initiate an immune response [51]. Several TLRs play important roles in the defense of different fish hosts, against various parasites e.g., *T. bryosalmonae* [52], *I. multifiliis* [53], *C. irritans* [54], and *C. rogercresseyi* [55]. TLR1, TLR2, TLR9, TLR19, TLR21, and TLR25 reportedly play an important role in immunity in fish against *I. multifiliis* [53]. However, the concerned parasite epitopes that they recognize are unexplored currently for the majority of TLRs. For example, TLR1, TLR13, and TLR19 are significantly upregulated in the kidney of brown trout during PKD [52], but the moiety of *T. bryosalmonae*, which might be responsible for their activation is unknown. TLR2 on human NK cells are activated by lipophosphoglycan component in the hemoflagellate parasite *Leishmania major*, a phosphoglycan belonging to a family of unique *Leishmania* glycoconjugates [56]. Phagocytic cells such as neutrophils, acidophilic granulocytes, and monocyte-macrophages, are involved in pathogen clearance [57] by producing reactive oxygen intermediates and nitric oxide, resulting in a respiratory burst. Increased phagocytic oxidative burst due to the enhanced capability of phagocytes to produce a higher amount of reactive oxygen species was observed in natural *T. bryosalmonae* infection in rainbow trout [58]. A higher number of NBT-positive cells indicated similar results in fish immunized with either *Sphaerospora dicentrarchi* spores alone or in combination with an adjuvant on the seventh day post-injection [59]. Respiratory burst and abundant granulocytes were found in vaccinated fish with live *Cryptobia salmositica* [60]. Furthermore, the increased number of thrombocytes and monocytes indicated clearance of *S. molnari* by phagocytosis. Increased thrombocytes acted against inflammation and contributed to wound healing [61]. Moreover, these authors associated the immediately increased expression of cytokines such as interleukin-1β upon peritoneal injection of the parasite with parasite recognition by host TLRs.

Nonspecific cytotoxic cells (NCC) in fish comprise heterogenous leucocyte populations. These are considered as mammalian equivalents of natural killer cells (NK) sharing many characters in common including antiparasitic activity [62]. NCC occur in teleost thymus, kidney, spleen and blood. They are particularly considered important in young fish when specific responses are weak [63]. The antiparasitic activity of NCC is reported against *I. multifiliis* [62] and *Tetrahymena pyriformis* [64]. However, more information on the role of these cells during parasitic infections is required.

In vitro studies with inactivated serum have reported the function of the classical complement pathway in killing *Philasterides dicentrarchi* [65]. Melanomacrophage centers (MMCs) are aggregation of phagocytic cells in lymphoid organs and involved in pathogen sequestration and antigen presentation [66]. MMCs have been found to contain different myxozoan parasites such as *Enterozoon scophthalami* [67] and *Myxobolus cyprini* [68]. The non-specific cellular innate immune response plays a greater role in the recovery from infection with parasites such as *T. bryosalmonae* [58]. The antiparasitic activity of different fish antiproteases such as α-2 macroglobulin during *E. scophthalmi* infection [69], total serum antiprotease in *E. leei* [67], and serine protein inhibitors [70] have been reported.

Only a few studies have been conducted to understand the susceptibility and resistance to parasites by host fish. For example, the upregulation of IFN-γ was reported both in the resistant Hofer strain and the susceptible US strain of rainbow trout against *M. cerebralis* infection. However, the upregulation of this gene was found to be considerably higher in susceptible trout strains than in the resistant strains after 24 h of pathogen exposure. The higher expression of the gene in susceptible rainbow trout was suggested to be deleterious for the host [71]. Similar observations in susceptible and resistant strains of Chinook salmon to *C. shashta* infection have been reported [72]. IFN-γ has been identified as a key immune effector cytokine with multiple protective roles via enhancing antigen presentation by APCs and initiation of pro-inflammatory responses in coordination with other pathways. Its activity is highly regulated, and its overexpression can damage the host tissue [48]. Among the different genes analyzed, STAT-3 (signal transducer and activator of transcription 3) was the most differentially expressed gene during *M. cerebralis* infection in susceptible and resistant rainbow trouts. In the Hofer strain rainbow trout, the higher expression of STAT-3 gene likely confers resistance against whirling disease. The expression remains unchanged in susceptible rainbow trout during infection [71]. SOCS (suppressor of cytokine signaling) protein family negatively regulates cytokine and growth factor signaling [73]. *T. bryosalmonae* and *M. cerebralis* infection results in the overexpression of SOCS-1 and SOCS-3 genes in rainbow trout during parasite development [74]. The innate and adaptive immune systems are interconnected. Therefore, the knowledge of innate immune effectors in protection, susceptibility, and resistance against parasites in fish is paramount in designing successful vaccines.

Some parasites reportedly evade or suppress the innate immune responses for their continued survival in the fish host. For instance, the ectoparasite *I. multifiliis* invokes a host evasion mechanism by ingestion of neutrophils, thus suppressing further signaling pathways in the immune reactions [75]. *L. salmonis* induces limited innate immune response in one of its most susceptible salmonid host Atlantic salmon which might be an immune suppression approach. Mustafa et al. [76] reported depression in oxidative and phagocytic abilities of host macrophage following *L. salmonis* infection which could be a parasite strategy. Another possible mechanism might be the increased production of proteases by the parasite. *L. salmonis* reportedly produces trypsin-like proteases which help them to feed and evade host immune responses. Dalvin et al. [77] reported the suppression of complement and nitric oxide in rainbow trout infected with sea lice. Nitric oxide is a signaling molecule leading to an anti-inflammatory response. It modulates the release of different inflammatory mediators from the cells participating in inflammatory responses, e.g., leukocytes, macrophages, mast cells, endothelial cells, and platelets [78]. Thus, suppression of nitric oxide could be a possible parasite strategy to avoid excessive immune response and increase survival of fish host. Additionally, *T. bryosalmonae* causes a severe inflammatory response in the kidney of brown trout which gradually regresses over a period of time and the recovered fish continue to excrete parasite spores [43].

### 4.2. Adaptive Immune Response during Selected Parasite Infection

Three classes of immunoglobulins have been described in fish, namely IgM, IgD, and IgT [79]. Parasite-specific antibodies have been detected in several parasitic infections, e.g., *T. bryosalmonae* [80,81], *M. cerebralis* [82], *S. dicentriarchi* [83], *C. shasta* [84], *E. scophthalmi* [85], *M. honghuensis* [86], *I. multifiliis* [75] and *L. salmonis* [77]. IgT might be playing important roles against fish parasites. In fish surviving *C. shasta* infection, a higher number of IgT^+^ B cells were found than in healthy fish [87]. The authors confirmed the IgT protein abundance and the upregulation of IgT gene. IgT is reportedly the dominant immunoglobulin present in skin and gills of *I. multifiliis*-infected rainbow trout. Abundant IgT^+^ B cells were found to occur in the skin epidermis of infected fish. Additionally, IgT was found to cover the parasite and was present in the mucus at high quantities [88]. All three immunoglobulins are formed in response to PKD; however, these might act as an escape mechanism following infection with *T. bryosalmonae*. This study also suggested an important role of IgD in the humoral response to the parasite, based on the appearance of IgD^+^ IgM^−^ B cells, somatic hypermutation, and clonal expansion of some IgD-expressing B cell subsets [89]. During *I. multifiliis* infection, antibodies cross-bind to Iag allowing the parasite to either escape from the host or result in destruction of the parasite [45]. During certain parasitic infections, antibodies were not detected. This response was observed in *C. elongatus*-infected Atlantic salmon because this parasite causes little damage to fish skin leading to decreased contact between louse antigen and the host immune system [90].

T cells are involved in adaptive immunity in all vertebrates. These cells are characterized by the presence of T cell receptors [91]. At present, studies on T cell-mediated immune response in fish parasites are mostly limited to the associated surface markers. Experimental infection by anal intubation of *E. leei*-infected intestinal scraping in gilthead seabream provided evidence for specific T cell response in the head kidney and anterior intestine [35]. In this study, real-time PCR revealed the downregulation of T cell markers such as zap70, cd3, cd4–1, cd4–2, cd8β, and CTL receptors in the head kidney and their upregulation in the anterior intestine. Moreover, FACS analysis provided evidence on the involvement of CD8^−^ T cells in resistance against *M. cerebralis* infection in the German Hofer strain of rainbow trout [92]. During infection with the intracellular parasite *K. thyrsites*, a Th 1 type response was reported as indicated by the upregulation of il-12 gene in the infected muscles of Atlantic salmon [33]. Currently, T cell responses are measured by ELISPOT assay, which is considered the most sensitive determination method of T cell cytokine production. Additionally, other assays are also used like intracellular staining for cytokines and others markers of T cell activation and function, in vivo CTL assay for measuring the lytic capacity of cytolytic T cells, which provide high sensitivity detection of specific pathogen peptides. These assays are useful in studying the specificity and potency of T cell responses of host and high throughput antigen screening [93]. Adaptive immune response mediated by high antibody titers with decreasing parasitemia was noticed following live vaccination against *C. salmositica* in Atlantic salmon [60]. Both the components of the adaptive immune system, i.e., the humoral response mediated by antibodies and the cellular response mediated by T cells are demonstrated in fish against parasitic infections. The humoral response is important against extracellular parasites, whereas T cell-mediated immune response is induced against intracellular parasites. Thus, an effective vaccination strategy would stimulate both these arms of the adaptive immune system [94].

## 5. Parasite Vaccines: Status and Prospects

Currently, there exist 24 commercially available vaccines for viral and bacterial diseases of aquaculture importance [16]. However, only one commercial parasite vaccine, Providean Aquatec Sea Lice produced by Tecnovax S.A. Argentina, is available. As discussed earlier, developing vaccines against aquatic parasites is an extremely challenging task.

Progress towards the development of parasite vaccines for aquaculture has been comparatively slower than those for humans and animals. In addition to the reasons mentioned, several other possibilities exist for this dismal scenario. A possible reason in several countries could be the smaller scale of aquaculture operations resulting in economic impracticality of vaccine production, the recent realization of their importance in causing huge disease losses, and fewer research groups working on this aspect. The available literature suggests that vaccines for parasites infecting fish are confined to a few trials against a limited number of parasites.

### 5.1. Ectoparasite Vaccine Trials

Vaccines have been attempted for parasites with significant detrimental effects on aquaculture. These include vaccination trials against parasites such as *I. multifiliis*, *C. irritans*, and *L. salmonis* (Table 2). Different vaccine trials comprising live, killed, parasite homogenate, subunit, and DNA have been tested for protection against *I. multifiliis* infection in various fish hosts. The earliest, as well as the most effective method of achieving protection to date remains immunization by live theronts or trophonts [30]. Immunization trials against this parasite with ciliary and whole cell preparation of *I. multifiliis* and *Tetrahymena pyriformis* were used to vaccinate channel catfish. The ciliary antigen of the latter was found to confer effective protection [95]. The lower protection rate obtained using *I. multifiliis* cilia could be attributed to less homogenous antigen preparation, resulting from the lower number of tomites used. The same fine structure of all protozoan cilia [96] and common antigenic determinant [97] provided cross protection. Burkart et al. [98] studied different antigen preparations and vaccination routes for immunizing channel catfish against *I. multifiliis*. The study concluded that intraperitoneal administration of live tomites effectively protected against infection as compared with a surface infection with the parasite. Fish vaccinated with formalin-killed trophonts resulted in a 51% mortality rate. Sonicated and formalin-killed trophont formulations protected rainbow trout ten weeks post-hatch fry. Moreover, this study reported a higher susceptibility to reinfection of bath-treated fish in comparison to IP-injected fish. However, the underlying reasons could not be ascertained [98]. Proteomic screening and in silico analysis were used to test three recombinant proteins (#5, #10, and #11), which were combined in a subunit vaccine and administered intraperitoneally in rainbow trout and resulted in partial protection. Parasite burden was found to be lower with a mean intensity of 1.3 parasites per g/cm fish in the vaccinated group as compared with the uninfected control group (2.8 parasites per g/cm fish).

In addition, specific antibody production was found to be significantly higher than in control fish. The highest antibody response was generated in fish against protein #10 both at four weeks and 13 weeks post-infection, suggesting it as a potential vaccine candidate along with cell and ciliary surface antigens commonly known as immobilization antigen or i-antigen [101]. For the DNA vaccine, the intramuscular administration of i-antigen alone or in combination with cysteine protease of *I. multifiliis* has been used in channel catfish and rainbow trout as a vaccine candidate [99,102]. However, the protection of DNA vaccine was comparatively lower than that of a live vaccine. Both live and killed *C. irritans* theronts conferred protective immunity in grouper, as evident from the high antibody titer in the mucus of immunized fish and better survival and reduced number of tomonts and trophonts [106].

### 5.2. Endoparasite Vaccine Trials

Currently, the literature on vaccination trials for endoparasites is available only for *T. bryosalmonae*, *Myxobolus koi*, and *Uronema marinum* (Table 3). DNA vaccine targeting a novel micro-exon genes (Tb MEG1) has been reported to elicit Tb-MEG1-specific immune response in rainbow trout [108]. Moreover, this study demonstrated the expression of proteins in and on the surface of parasites using anti-Tb-MEG1 monoclonal antibodies. A study using crude spore proteins reported better survival in *M. koi*-infected gold fish [109]. Intraperitoneal administration of poly D, L-lactic-co-glycolic acid (PLGA)-encapsulated vaccine against *U. marinum* infection significantly reduced the mortality in kelp grouper, *Epinephelus bruneus* [110]. This study reported early enhancement (1 to two weeks post-vaccination) and longer duration (4 weeks post-vaccination) of respiratory burst, complement activity, α2-macroglobulin activity, serum lysozyme, antiprotease activity, and antibody response. Studies on vaccination trials for ecto- and endoparasites report the use of different criteria for determining the efficacy of the tried vaccine, such as percentage mortality, percentage survival, parasite burden, and antibody titer in vaccinated fish as compared with those in the control fish.

## 6. Perspectives in Fish Parasite Vaccine Development

### 6.1. Vaccination Strategy

Live, killed, DNA and protein subunit vaccines are being applied in aquaculture to control various pathogenic organisms. The majority of the vaccine trials in fish against parasites have used the live or killed strategy that has proved effective. However, it has certain limitations. Live vaccination is similar to the natural process of infection. This approach employs a controlled infection with a virulent or less virulent or attenuated strain of the parasite. In the case of fish, the attenuated strains of few parasites has been used for immunizing fish. There is an increased risk of mortality with vaccination with live virulent parasites. Another important consideration for both live and killed vaccines is the requirement of numerous parasites of good quality, which currently is difficult to culture in vitro. In addition, live vaccines are not considered safe for use in aquaculture [16]. Although DNA and subunit vaccines are the most promising alternative approaches, both the approaches have conferred partial to moderate protection. This could be attributed to the fact that the parasites present several challenges such as antigenic variability, immune evasion, immunomodulation of effector molecules, and poor immunogenicity of individual antigens. To overcome these issues, the vaccine must target multiple antigens (multi-epitope) simultaneously to be effective. Although a successful example of a multivalent vaccine approach for parasite is not available from fish-based trials, a multivalent DNA vaccine encoding three antigens provided long-lasting protection after mice were challenged with *Leishmania* [112]. Certain factors must be considered while attempting a subunit vaccine, including the technical viability of antigen production, its formulation in suitable adjuvants, and the ease and frequency of delivery [113]. Similarly, the selection of expression vector must ensure the required post-translational processing to retain the immunogenicity of the desired protein.

### 6.2. Vaccination Routes

Broadly, vaccines can be administered in aquaculture via oral, immersion, and injection routes. In the oral process, antigens encapsulated in the feed are administered to fish. It is the most effective delivery method for aquaculture due to minimal stress, simple administration, and applicability to both large and small fish. However, difficulty in determining the precise dosage received by fish and lack of efficacy limit its application. In addition, antigen degradation is possible during its passage to the stomach before it reaches the hindgut where the antigen is adsorbed [114]. In the immersion process, fish are immersed in water with antigens for a specified period. Immersion can be performed in the form of dip and bath. In the dip method, the fish are kept in water containing antigens at a high dose for some minutes, whereas the bath method involves keeping the fish in water with a low antigen dose for a longer time [115]. The injection route is used to commonly deliver the antigen either intraperitoneally or intramuscularly. The advantages of injection vaccination include precise dosage delivery and longer protection [116]. In most studies, the intraperitoneal route of vaccine administration against parasites was used for live and killed antigen preparations, whereas the intramuscular route has been used for DNA antigens (Table 2 and Table 3). Several studies report significant systemic and mucosal antibody production against parasites following the intraperitoneal injection of antigens [98,101]. Certain disadvantages associated with the injection process include stress during handling and anesthetizing. In addition, the method is labor intensive, costly, and impractical for fish below 20 g and for mass vaccination [117].

### 6.3. Protective Immune Response

The success of any vaccine against a parasite depends on the development of protective immunity in the fish host. The developed protective immune response should mimic as during a naturally occurring parasitic infection. Furthermore, the magnitude of immune response depends on the pathogen, intensity, and the stage of infection [118]. The protective immune responses of fish against some parasites are dependent on the production of specific antibodies. For example, specific antibodies were found in the sera of recovered and experimentally infected fish, which were confirmed to involve in protective immunity in fish against *Cryptobia salmositica* and Scuticociliates [119,120]. Anti-*T. bryosalmonae* specific antibodies have been detected in sera of experimentally infected brown trout from 4 to 17 weeks post exposure; however, studies on the mechanisms involved in protection are limited (authors own unpublished data). Partial antibody-mediated protection against *I. multifiliis* and *Trypanosoma carassii* was obtained after immunization of fish with recombinant proteins [121,122]. However, in several cases, the presence of specific antibodies were not correlated with protection of fish against parasites.

In the context of fish parasites, information on specific parasite stages that elicit immune response is mostly available for ectoparasites. For example, immunity is directed against the theront and trophont stages of *I. multifiliis* [123] and *C. irritans* [124]. Additionally, from metanauplius larvae to adult *Argulus* stages induce limited host response [18]. With regard to myxozoan endoparasites, the knowledge of host response on intrapiscine development of myxospores exists; however, specific stages capable of eliciting immune response is unknown. In cases of parasites exploiting the mucosal surface to invade the host, such as the myxozoans, the ectoparasites such as *I. multifiliis* and *C. irritans*, the hosts counter the invasion by initiating a mucosal immune response. Therefore, the prophylactic vaccines for such pathogens should be designed to stimulate mucosal immunity. Humoral components such as complement impart protection against *P. dicentrarchi* infection in turbot [125]. Both B cell and T cell responses confer protection in several infections, which depends on both the host and the parasite. Therefore, a vaccine for controlling *P. dicentrarchi* should activate the complement, whereas in the latter case should result in B cell- and T cell-mediated responses. Vaccines for fish parasites, e.g., *E. leei* and *K. thyrsites*, should induce T cell-mediated immune response, using vectors such as plasmid DNA or viruses [126]. Vaccines targeting secretory enzymes have shown promising results against the hookworm *Ancyclostoma caninum* [127] and the helminth *Schistosoma mansoni* [128]. A similar approach can be followed for the crustacean parasites e.g., *L. salmonis*, *C. elongatus* and *Argulus* which use many secretions as their immunesuppression strategy. Moreover, the nature of adjuvants affects the desired immune response. For example, cell-mediated immune response can be achieved by the administration of cytokines along with antigen [129] or by heterologous prime-boost approach, wherein the antigen is delivered sequentially using different vaccine platforms [130]. Oil-based adjuvants generate a high antibody titre in blood [113]. Another important aspect of consideration for a successful vaccination is the vaccine delivery, including the mode of administration, the dosage, and the timing.

### 6.4. Long-Term Immunity

Immunological memory underpins the concept of long-term immunity and vaccination. Vaccination-induced immune memory provides antibodies continuously and maintains memory cells to allow rapid response on exposure to the pathogen. The existence of immunological memory has been demonstrated in fish [131]. However, studies on its duration in response to parasitic infections in fish are limited. An epidemiological investigation on PKD in rainbow trout reported the presence of immunity in surviving fish during the subsequent year after infection [132]. Following exposure to theronts, channel catfish were found to be immune to re-infection after 3 years due to activation of memory B cells and mobilization of Iag-specific antigen-secreting cells into both systemic and mucosal compartments [133]. T cell-mediated immune response has been demonstrated during certain parasitic infections, e.g., *T. bryosalmonae* [134], *E. leei* [35], and sea lice [135]. In vertebrates, during the natural course of infection, following its control, the pathogen load along with T cell declines. The T cell survivors are responsible for immunological memory. Therefore, the extent of expansion and contraction along with the resulting memory depends on several factors such as the type and amount of antigen, the duration of antigen exposure, the site of antigen introduction, and the ability of the antigen or its co-delivered components to activate innate immune responses [93].

## 7. Challenges in Vaccine Development

Lack of knowledge of biology and the life cycle of parasites are the biggest challenges in developing vaccines against them. Although the life cycle aspects of a few parasites are relatively well understood [93,135], it is unknown for the majority of the fish parasites. Consequently, the cultivation and maintenance of parasites under laboratory conditions becomes difficult; however, it is a necessity for the preparation of antigens and the production of antibodies for vaccine development [136].

Another limitation is the immune escape mechanism of parasites that allows them to evade the direct interaction with the host’s immune system. Fish parasites have devised different strategies to escape the host immune defense during infection. For example, *M. cerebralis* invades the nerves, which is an immunologically privileged site with low host immune response [137]. Antigenic variation is an important feature of protozoan parasites that enables them to evade the host immune response and leading to chronic infections [136]. Antigenic variations of *I. multifiliis* have been confirmed by Northern hybridization, suggesting the expression of genes coding immobilization antigens in different life stages of *I. multifiliis* [138]. In addition, antigenic variations, reflected by nine putative I-antigens, have been reported in *C. irritans* from different life stages (tomont, theront, and trophont) using transcriptomic analysis [124]. Further, vaccine development is challenged by poor information on host–parasite interaction and immune response. As an example, although TLRs are known to play an important role in host defense against pathogens, including parasites, the ligand specificity is not yet determined for the majority of the TLRs. In addition, no information is available on different populations of cells in fish expressing TLRs. Such knowledge holds immense importance in the understanding of the resistance mechanisms in fish against parasites, and the development of novel adjuvants and more effective vaccines [139].

Presently, the lack of knowledge of specific antigens of parasites, which trigger the protective immune response is a major constraint. Large-scale production of recombinant proteins that retain the immunological activity similar to the natural parasite protein is an additional roadblock for the development of recombinant vaccines.

## 8. Role of Omics Technologies in Vaccine Development

Significant research in the study of parasites of human and veterinary importance highlight the potential of omics in the development of vaccines. Analysis of data obtained from omics-studies are an important source of information on SNPs, resistance markers, changes in gene expression, splicing variants, protein modifications, and strain-specificities of parasites. Such information helps in understanding biological attributes of the parasite that may aid in developing disease control strategies [140]. For example, characterization of proteins encoded by the polymorphic loci detected from genomic data of *Plasmodium falciparum* [141], can help in identification of vaccine targets [140]. Hypervariability of parasite antigens is viewed as a major obstacle in developing vaccine against them. Alternative splicing of parasite surface proteins is an important phenomenon that may result in isoforms differing in cell localization, substrate affinities and functions. The structural differences of the isoforms can be large enough to enable the parasite to evade host-immune recognition [142]. RNA-seq based transcriptomic studies have elucidated the role of alternative splicing during cellular differentiation in *Plasmodium berghei* [143]. The majority of the proteins that play pivotal roles in invasion are either stored in the apical secretory organelles or located on the surface of the merozoite, the invasive stage of the *Plasmodium* [144]. Novel secretory organelle proteins and surface-exposed proteins were identified from proteomic analysis of *P. falciparum* [145]. Additionally, an integrated transcriptomic and proteomic approach was applied to describe the *Fasciola hepatica* secretory proteome, thus identifying proteins such as cathepsin, peroxiredoxin, glutathione S-transferase, and fatty acid-binding proteins essential for the design of the first-generation anti-fluke vaccines and flukicidal drugs.

In view of the commonalities existing between mammalian and aquatic parasites (e.g., life cycle with multiple stages, multiple hosts and stage specific antigen profile) and considering the fact that research in mammalian parasitology is far ahead of aquatic parasitology, important lessons need to be learnt from both their failures and successes. The human endoparasites such as *Plasmodium*, *Leishmania* and *Trypanosoma* have been the focus of extensive research for many decades. It has been established by now that early efforts to develop effective vaccines against these parasites have failed as a result of a poor selection of few antigens without the knowledge of antigen repertoire of the parasites [146]. Past efforts have relied on single gene, transcript or protein in vaccine formulations. Likewise, as discussed earlier, vaccination trials against fish parasites have focused on either single or dual target vaccines resulting in limited or suboptimal protection. However, evidences from laboratory experiments [147] and field studies [148] on *Plasmodium* uphold the requirement of a multivalent vaccine wherein a robust immune response towards multiple antigenic determinants can be elicited to provide optimal protection the in host. A multivalent vaccine against human visceral leishmaniasis reportedly elicited significant humoral and cellular responses in pre-clinical trials [149]. Another multivalent vaccine containing stage-specific antigens of *Fasciola hepatica* conferred 83% protection in vaccinated rats [150]. Drawing from these studies from mammalian parasites, it is likely that a multivalent approach might be a better strategy against both endo- and ectoparasites of fish.

In fisheries and aquaculture, the application of omics has just begun, triggered by the advancement as well as cost reduction in NGS technologies [151]. Of the many omics techniques, the application of transcriptomics, genomics and proteomics are emerging in fish parasitology.

### 8.1. Transcriptomics

Transcriptomics-based studies have widely been used to explore the viral and bacterial pathogen–fish interactions [24]. Similar studies focusing on parasitic diseases in fish are limited. Yet, these restricted works on parasite-induced pathologies have elucidated important phenomena related to the host, such as immune response and concerning the parasite, possible escape strategies and functional biology. Recently, an RNA sequencing-based transcriptome study provided valuable insights into the immune response mounted by brown trout host, as well as modulated host machineries in response to *T. bryosalmonae* infection [152]. Additionally, transcriptome of bryozoan *Fredericella sultana* has been demonstrated, which provides valuable resources for the understanding of the unique biological characteristics and functional transcripts of this important bryozoan species that is the primary host of *T. bryosalmonae* [153]. Transcriptome analysis of *T. bryosalmonae*-infected *F. sultana* revealed 1166 differentially expressed genes with Eukaryotic Initiation Factor 2 signaling as a top canonical pathway and MYCN as a top upstream regulator [154]. Furthermore, the transcriptome of *T. bryosalmonae* from *F. sultana* is found to contain several members of the protease family, e.g., cathepsin L, cysteine protease, zinc metalloprotease, and serine protease [155]. The transcriptome analysis of *E. scophthalmi*-infected turbot suggested the role of IFN-mediated signaling pathways during incipient enteromyxosis as well as the downregulation of complement and acute phase proteins as possible immune evasion mechanisms [156]. De novo assembled transcriptome of *Sphaerospora molnari* blood stages are reported to contain 9436 proteins. This work has provided valuable information on a proteolytic depot of the parasite consisting 235 putative proteases, mainly of cysteine proteases [157]. Furthermore, genome and transcriptome-based data analysis of *Kudoa iwatai* and *M. cerebralis* was used to study the evolution of endoparasitism in myxozoans [158].

The comparative transcriptomic profile analysis of trophont, tomont and theront stages of *C. irritans* haselucidated the differentially expressed genes with functions in cell division, nutrition analysis and cell growth. Moreover, nine putative immobilization antigen (I-antigen) and protease transcripts, which can be considered as potential vaccine and drug targets were also found [159]. *Argulus siamensis* transcriptome examination revealed the presence of serine and metalloprotease that are known antigens in many ectoparasites. The study also led to the characterization of the downward signaling molecules of toll pathway [31].

### 8.2. Proteomics and Genomics

Proteomics and genomics are the other modern high-throughput techniques that have become instrumental in exploring different aspects of both the host and pathogen separately and their interactions at the molecular level. Kumar et al. [160] identified host–parasite protein interaction during proliferative kidney disease using antibody-based purification followed by electrospray ionization mass spectrometry. These identified proteins can be used for understanding the pathogenesis and defense mechanisms of *T. bryosalmonae*. Piriatinskiy et al. [161] identified polar capsule proteins of *C. shasta* using tandem mass spectrometry and suggested that polar capsules and nematocysts are homologous organelles. This study unraveled 112 proteins present in polar capsules of *C. shasta* with diverse functions such as the structural (nematogalectin and minicollagen), protein folding (HSP 70 and isomerases) enzymes involved in poly-γ-glutamate biosynthesis in addition to some novel proteins containing cysteine-rich and proline rich stretches.

Nano-LC ESI MS/MS based proteome analysis of *I. multifiliis* infected fish skin mucus revealed the involvement of innate immune components, e.g., lectins and serpins, in providing protection against the parasite [162]. An iTRAQ (Isobaric tags for relative and absolute quantitation) based quantitative proteomic study identified 2300 proteins in theront and trophont stages of *I. multifiliis*, of which 1520 proteins were differentially expressed in trophonts. These proteins played important roles of binding, catalytic activity, structural molecule activity and transporter activity in the parasite life-cycle [163]. The comparative proteomic analysis of theront, trophont, and tomont of *C. irritans* using 2D-gel electrophoresis and mass spectrophotometry identified different proteins, which could be used as vaccine candidates. Among these, α-tubulin and actin were found to be expressed in all the three developmental stages, whereas enolase was present in theront and trophont and vacuolar ATP synthase (V-ATPases) catalytic subunit α was detected only in theronts [163].

Genome sequencing and analysis of the transcriptionally active macronucleus of *I. multifiliis* has revealed several gene classes functioning in behavior, cellular functions, and host immunogenicity, including protein kinases, membrane transporters, proteases, and surface antigens, providing avenues for selecting vaccine and drug targets [164]. These gene families are identified as lead vaccine targets in many parasites of medical and veterinary importance. For example, members of the protein kinase family, e.g., *Toxoplasma gondii* calcium-dependent protein kinase 2 [165] and proteases [166] are considered promising vaccine candidates against the apicomplexan parasite *Toxoplasma gondii* that infects warm blooded animals [165]. Proteases are excellent shistostome vaccine targets as well. Membrane transport proteins are amongst the most attractive molecular targets of FDA-approved drugs [167].

Overall, the unprecedented wealth of information generated by the analysis of genomic, transcriptomic and proteomic data can better our knowledge of the host as well as the parasite. As discussed, omics can be significant in the identification of suitable antigen candidates of parasites; as such, it is important to note that the efficacy of previously reported vaccines can also be increased. This can be achieved by combining the already reported antigens with the ones selected based on omics data. For example, Jorgensen et al. [75] reported the immunogenicity of the recombinant protein #10; however, it is also noted that in combination with other suitable candidates, it can lead to better protection in fish on vaccination.

Additionally, advanced genome-based techniques can be significant in overcoming many issues concerning complex parasitic organisms, one of the important hurdles being the requirement of parasite culture. This is because maintaining a parasite life-cycle necessitates the maintenance of intermediate and final hosts, which are costly and exhaustive procedures requiring a large amount of time and effort. This is followed by antigen identification, isolation, and purification, which further involves several challenging steps. Besides, omics approaches will possibly be advantageous in cases where many genes and proteins are expressed only during the course of infection and where biomolecules are present in insufficient quantities to be recognized by assays. To date, sequence data are available for a limited number of ectoparasites and endoparasites (Table 4).

## 9. Approach of Multivalent Vaccines

**Figure 1:**
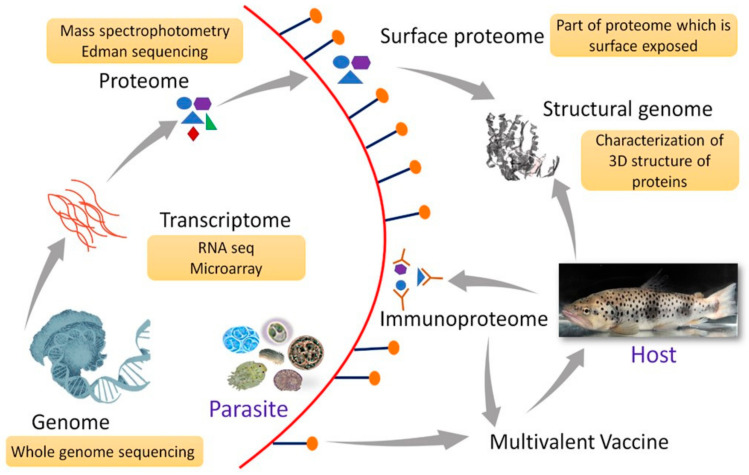
Conceptual representation of multivalent/multiepitope vaccine formulation for fish parasites in the omics era. Analysis of genomic, transcriptomic and proteomic data of parasite and the data obtained from host–parasite interactions enable identifying a suitable antigen as vaccine candidates. Genome sequence contains the entire genetic repertoire of antigens from which novel vaccine targets can be selected. Transcriptome analysis provides insights on the parasite gene expression profile leading to successful establishment and pathology in host. Proteomic analysis provides information on protein expression under specified conditions. It is useful in identification of proteins that are expressed by parasite during infection and the subset of proteins which are present on parasite surface (surface proteome). Surface exposed proteins which are immunogenic in the host can be suitable vaccine candidate. Structural genomics helps to know the three-dimensional structure of proteins produced by an organism and how they interact with antibodies or drugs. Immunoproteomics provides information on the proteins or epitopes which interact with host antibodies.

The insights gained from omics data can be invaluable in the development of multivalent vaccines. A multivalent vaccine is a combination of several antigens to elicit a broad protective immune response by the host [173]. The different antigens can be selected from the same parasite, different parasites, parasite strains, and developmental stages. Considering the multiple critical problems associated with the aquatic parasites, particularly the diverse antigenic profile of the developmental stages and parasite strains, a multivalent vaccine, in general, would be an effective strategy against them. For certain parasites like *T. bryosalmonae*, although cell-mediated immune response is speculated to protect fish against infection [58], the exact protective response is not yet defined. Under these circumstances, multiple antigenic candidates can be selected and targeted based on studies on the parasite transcriptome, genome, and proteome along with host–parasite interaction data. Furthermore, a study with inactivated vaccine containing two different isolates of *P. dicentrarchi* did not confer cross-protection in turbot [65]. This could be explained by the fact that the parasite exhibits intraspecific variation both at the morphological and genetic levels [174]. The development of vaccines for such parasites would entail a pan-genomic approach wherein antigens conserved across genomes can be the target for a broad spectrum of protection. A conceptual representation of the multivalent vaccine using an omics dataset is presented in Figure 1.

**Figure 2:**
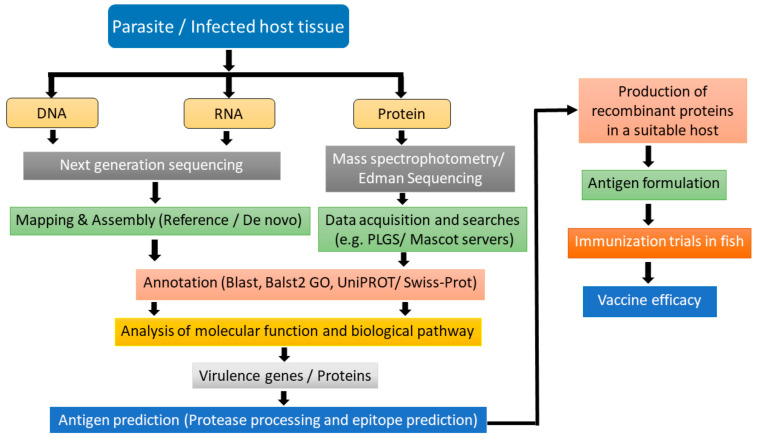
Workflow for identification of vaccine candidates and vaccine formulation using high throughput omics techniques. The material of interest (DNA, RNA or protein) can be extracted from the desired parasite or from-infected host fish tissue. The isolated material serves as sample for next generation sequence analysis (for DNA and RNA) and spectrophotometry and Edman sequencing (for protein). The obtained sequences can be subjected to different bioinformatic tools for analysis and functional annotation of parasite molecules. Based on molecular function and biological pathway analysis, the immunogenic targets (capable of eliciting host immune response) can be selected and their protective epitopes predicted. These molecules can then be produced on a large scale and combined with suitable adjuvants to form vaccine. Subsequently, the vaccine thus produced can be used for conducting trials in suitable fish and its efficacy and appropriate route of administration can be determined.

In designing a multivalent vaccine, genomics, transcriptomics, structural genomics, proteomics, and immunoproteomics can be used to identify a suitable antigen. An approach for the selection of antigenic candidates and the development of vaccines utilizing omics approaches is presented in Figure 2. In this approach, whole-genome sequencing, RNA sequencing, and proteomics data can be used as a starting material of the targeted parasite or the infected host tissue. Further annotation of the data can be carried out using different bioinformatics tools such as Blast, Blast2GO, and UniProt/Swiss-Prot. After annotation, the gene, RNA transcripts, and proteins can be analyzed for their function as well as the different biological pathways. Vaccine candidates can be administered in fish to check the efficacy and potency of the vaccine.

## 10. Conclusions

There has been a recent increase in the outbreak of parasitic diseases in farmed and wild fish populations. The general practice for controlling parasitic infestations is to use chemotherapy. Over the past few decades, several concerns have been raised regarding their use, including environmental safety. The experts have recommended vaccines as effective solution to address these issues. Different types of vaccines have shown varying degrees of protection in fish against parasites. However, deeper insights into the host–parasite interaction and parasite’s life cycle with different stages are needed to be overcome for the development of successful vaccines. Research on some ectoparasites have been focused with the aim of developing vaccines. However, little attention has been given to the endoparasites. As an example, very little is known about the genes of myxozoans that are induced and expressed in both hosts (invertebrates and vertebrates) and involved in pathogenicity in the fish host during the course of infection. Hence, the identification of the in vivo induced genes of parasites related to disease development is required to improve our understanding of pathogenesis and promote the discovery of novel therapeutic targets. At present, transcriptome, genome, and proteomic data are limited for fish parasites. However, there is a need of large amount of omics data pertaining to more fish parasites for the identification of potential vaccine candidates and designing multivalent vaccines.

## Figures and Tables

**Table 1:** Some economically important fish parasites: Table provides information on mortality and economic loss caused by important parasites of fish. NA: not available, a: mortality, b: economic loss.

Parasite Group	Parasite	Disease	Host	Mortality ^a^/ Economic Loss ^b^	Reference
		Freshwater Endoparasites			
Cnidaria	*T. bryosalmonae*	Proliferative kidney disease	Rainbow trout	95% ^a^	[26]
	*M. cerebralis*	Whirling disease	Rainbow trout	90% ^a^	[27]
	*S. molnari*	Sphaerosporosis	Carps	NA	[28]
Platyhelminthes	*Bothriocephalus acheilo* *gnathi*	Ulcer, catarrhal enteritis	Carps	100% ^a^	[29]
Freshwater Ectoparasites					
Ciliophora	*I. multifiliis*	White spot	Rainbow trout, Carps	NA	[30]
Arthropoda	*Argulus*	Argulosis	Indian Major Carps	5.41 million USD ^b^	[31]
Platyhelminthes	*Gyrodactylus salaris*	Gyrodactylosis	Atlantic salmon	NA	[32]
Marine Endoparasites					
Cnidaria	*K. thyrsites*	Soft flesh syndrome	Salmonids	6 million CAD ^b^	[33]
	*C. shashta*	Ceratomyxosis	Salmonids	NA	[34]
	*E. leei*	Enteromyxosis	Sharpsnout seabream	NA	[35]
Ciliophora	*Uronema marinum, Philasterides dicentrarchi*	Scuticociliatosis	Seabass, Turbot	NA	[36]
Marine Ectoparasites					
Ciliophora	*C. irritans*	White spot	Greater Amberjack	834 USD ^b^	[3]
Arthropoda	*Caligus rogercresseyi, Lepeophtheirus salmonis*	Sea louse disease	Salmonids	100 million USD ^b^	[37]

**Table 2:** Overview of immunization trials in fish for ectoparasites: Table provides information on immunization trials for important ectoparasites of fish. IM: Intramuscular, IP: Intraperitoneal.

Disease	Parasite	Fish Host	Vaccine Trial Type	Antigen Target	Delivery Method	Reference
White spot disease (Ich)	*Ichthyophthirius multifiliis*	Channel catfish	DNA	(i-antigen) immobilization antigen	IM	[99]
		Channel catfish	Live	Theront	IP	[100]
		Rainbow trout	Subunit	Recombinant proteins (#5, #10, & #11)	IP	[101]
			DNA	(i-antigen) immobilization antigen & Cysteine protease	IM, Needle free injection, Gene gun delivery	[102]
			Live	Theronts	IP	[103]
			Killed	Sonicated formalin killed trophonts	IP	[104]
		Nile Tilapia	Live	Theront and sonicated trophonts	Immersion and IP	[105]
		Channel catfish	Live	Tomites	IP	[98]
			Killed	Trophonts and tomites	IP	
Marine White spot	*Cryptocaryon irritans*	Grouper	Live	Theronts	Bath	[106]
			Killed	Formalin-killed theronts	IP	[107]
Sea louse infestations	*Lepeophtheirus salmonis*	Atlantic salmon	Crude parasite extract	Adult female parasite	IP	

**Table 3:** Overview of immunization trials in fish against parasites: Table provides information on immunization trials for important endoparasites. NA: Not Available, IM: Intramuscular, IP: Intraperitoneal.

Disease	Parasite	Fish Host	Vaccine Type	Antigen Target	Delivery Method	Reference
Freshwater Endoparasites						
PKD	*Tetracapsuloides bryosalmonae*	Rainbow trout	DNA	Micro-exon gene (TB-MEG1)	Not Available	[108]
Myxobolosis	*Myxobolus koi*	Gold fish	Subunit	Crude protein spore	Immersion	[109]
Marine Endoparasites						
Scuticociliatosis	*Uronema marinum*	Grouper	Subunit	(i-antigen) immobilization antigen	IP	[110]
	*Philasterides dicentrarchi*	Turbot	Subunit	Membrane proteins	IP	[111]

**Table 4:** Available sequence data for parasites: Table enlists the fish parasites for which genome and transcriptome data are available. a: Transcriptome size, b: Genome size, c: Number of contigs, NA: Not Available.

Parasite	Molecular Data Type	Sequencing Platform	Size/No. of Contigs	Accession No.	Reference
Endoparasite					
*Tetracapsuloides bryosalmonae*	Transcriptome	NextSeq 550	25908 ^c^		[155]
*Myxobolus cerebralis*	Transcriptome	HiSeq 2000	52972 ^c^	GBGI00000000	[158]
*Kudoa iwatai*	Transcriptome	HiSeq 2000	1637 ^c^	GBKL00000000	[158]
*Ceratomyxa shasta*	Transcriptome	Illumina HiSeq 3000	NA	SRX3741971	[168]
*Sphaerospora molnari*	Transcriptome	Illumina HiSeq	29560 ^c^	PRJNA522909	[157]
*Myxobolus squamalis*	Transcriptome	Illumina HiSeq 3000	NA	SRX4615721	[169]
Ectoparasite					
*Gyrodactylus salaris*	Whole genome	Roche 454 FLX Titanium Illumina GAII	120 Mb ^b^	JJOG00000000	[170]
*Cryptocaryon irritans*	Transcriptome (Trophont)	Illumina HiSeq 2000	2.6 Gb ^a^	SRX2417163	[171]
	Transcriptome (Theront)	Illumina HiSeq 2000	3.2 Gb ^a^	SRX2417144	[172]
*Argulus siamensis*	Transcriptome	Illumina HiSeq 2000	46352 ^c^	SRX150806	[31]

## Supplementary Materials

The following are available online at https://www.mdpi.com/2076-393X/9/2/179/s1, Figure S1: PRISMA flow diagram of study search and selection.

## References

[1] FAO (2020). The State of World Fisheries and Aquaculture 2020. Sustainability in Action.

[2] Murray A.G., Peeler E.J. (2005). A framework for understanding the potential for emerging diseases in aquaculture. Prev. Vet. Med..

[3] Shinn A.P., Pratoomyot J., Bron J.E., Paladini G., Brooker E.E., Brooker A.J. (2015). Economic costs of protistan and metazoan parasites to global mariculture. Parasitology.

[4] Yokoyama H. (2003). A review: Gaps in our knowledge on myxozoan parasites of fishes. Fish Pathol..

[5] Hoffman G.L. (1990). *Myxobolus cerebralis*, a worldwide cause of salmonid whirling disease. J. Aquat. Anim. Health.

[6] Bravo S., Sevatdal S., Horsberg T.E. (2008). Sensitivity assessment of *Caligus rogercresseyi* to emamectin benzoate in Chile. Aquaculture.

[7] Sevatdal S., Copley L., Wallace C., Jackson D., Horsberg T.E. (2005). Monitoring of the sensitivity of sea lice (*Lepeophtheirus salmonis*) to pyrethroids in Norway, Ireland and Scotland using bioassays and probit modelling. Aquaculture.

[8] Boxall A.B.A. (2004). The environmental side effects of medication: How are human and veterinary medicines in soils and water bodies affecting human and environmental health?. EMBO Rep..

[9] Johnson A.C., Jürgens M.D., Williams R.J., Kümmerer K., Kortenkamp A., Sumpter J.P. (2008). Do cytotoxic chemotherapy drugs discharged into rivers pose a risk to the environment and human health? An overview and UK case study. J. Hydrol..

[10] Valladão G.M.R., Gallani S.U., Pilarski F. (2015). Phytotherapy as an alternative for treating fish disease. J. Vet. Pharmacol. Ther..

[11] Ekor M. (2014). The growing use of herbal medicines: Issues relating to adverse reactions and challenges in monitoring safety. Front. Neurol..

[12] Yokoyama H., Ogawa K., Wakabayashi H. (1990). Chemotherapy with fumagillin and toltrazuril against kidney enlargement disease of goldfish caused by the myxosporean *Hoferellus carassii*. Fish. Pathol..

[13] Morris D.J., Adams A., Smith P., Richards R.H. (2003). Effects of oral treatment with TNP-470 on rainbow trout (*Oncorhynchus mykiss*) infected with *Tetracapsuloides bryosalmonae* (Malacosporea), the causative agent of proliferative kidney disease. Aquaculture.

[14] Flamarique I.N., Gulbransen C., Galbraith M., Stucchi D. (2009). Monitoring and potential control of sea lice using an LED-based light trap. Can. J. Fish. Aquat. Sci..

[15] Skilton D.C., Saunders R.J., Hutson K.S. (2020). Parasite attractants: Identifying trap baits for parasite management in aquaculture. Aquaculture.

[16] Adams A. (2019). Progress, challenges and opportunities in fish vaccine development. Fish Shellfish Immunol..

[17] Villegas A. First Ever Sea Lice Vaccine Launched in Chile. https://www.undercurrentnews.com/2015/11/24/first-ever-sea-lice-vaccine-launched-in-chile/.

[18] Woo P.T.K., Buchmann K. (2012). Fish Parasites:Pathobiology and Protection.

[19] Hasin Y., Seldin M., Lusis A. (2017). Multi-omics approaches to disease. Genome Biol..

[20] Kavallaris M., Marshall G.M. (2005). Proteomics and disease: Opportunities and challenges. Med. J. Aust..

[21] Hegde P.S., White I.R., Debouck C. (2003). Interplay of transcriptomics and proteomics. Curr. Opin. Biotechnol..

[22] Lowe R., Shirley N., Bleackley M., Dolan S., Shafee T. (2017). Transcriptomics technologies. PLoS Comput. Biol..

[23] Feder M.E., Walser J.C. (2005). The biological limitations of transcriptomics in elucidating stress and stress responses. J. Evol. Biol..

[24] Sudhagar A., Kumar G., El-Matbouli M. (2018). Transcriptome analysis based on RNA-Seq in understanding pathogenic mechanisms of diseases and the immune system of fish: A comprehensive review. Int. J. Mol. Sci..

[25] Feist S.W., Longsaw M., Woo P.T.K. (2006). Phylum myxozoa. Fish Diseases and Disorders.

[26] Clifton-Hadley R.S., Richards R.H., Bucke D. (1986). Proliferative kidney disease (PKD) in rainbow trout *Salmo gairdneri*: Further observations on the effects of water temperature. Aquaculture.

[27] Rognlie M.C., Knapp S.E. (1998). *Myxobolus cerebralis* in *Tubifex tubifex* from a Whirling Disease Epizootic in Montana. J. Parasitol..

[28] Lom J., Dyková I., Pavlásková M., Grupcheva G. (1983). *Sphaerospora molnari* sp.nov. (Myxozoa:Myxosporea) an Agent of Gill, Skin and Blood Sphaerosporosis of Common Carp in Europe. Parasitology.

[29] Körting W. (1975). Larval development of *Bothriocephalus* sp. (Cestoda: Pseudophyllidea) from carp (*Cyprinm carpio* L.) in Germany. J. Fish Biol..

[30] Matthews R.A. (2005). *Ichthyophthirius multifiliis* fouquet and ichthyophthiriosis in freshwater teleosts. Adv. Parasitol..

[31] Sahoo P.K., Kar B., Mohapatra A., Mohanty J. (2013). De novo whole transcriptome analysis of the fish louse, *Argulus siamensis*: First molecular insights into characterization of Toll downstream signalling molecules of crustaceans. Exp. Parasitol..

[32] Bakke T.A., Cable J., Harris P.D. (2007). The Biology of Gyrodactylid Monogeneans: The “Russian-Doll Killers”. Adv. Parasitol..

[33] Braden L.M., Rasmussen K.J., Purcell S.L., Ellis L., Mahony A., Cho S., Whyte S.K., Jones S.R.M., Fast M.D. (2018). Acquired protective immunity in Atlantic salmon *Salmo salar* against the myxozoan *Kudoa thyrsites* involves induction of MHIIβ+ CD83+ antigen-presenting cells. Infect. Immun..

[34] Kent M.L., Margolis L., Whitaker D.J., Hoskins G.E., McDonald T.E. (1994). Review of Myxosporea of importance in salmonid fisheries and aquaculture in British Columbia. Folia Parasitol..

[35] Piazzon M.C., Estensoro I., Calduch-Giner J.A., Del Pozo R., Picard-Sánchez A., Pérez-Sánchez J., Sitjà-Bobadilla A. (2018). Hints on T cell responses in a fish-parasite model: *Enteromyxum leei* induces differential expression of T cell signature molecules depending on the organ and the infection status. Parasites Vectors.

[36] Piazzon M.C., Leiro J., Lamas J. (2014). Reprint of “Fish immunity to scuticociliate parasites”. Dev. Comp. Immunol..

[37] Johnson S.C., Treasurer J.W., Bravo S., Nagasawa K., Kabata Z. (2004). A review of the impact of parasitic copepods on marine aquaculture. Zool. Stud..

[38] Jones S.R.M. (2001). The occurrence and mechanisms of innate immunity against parasites in fish. Dev. Comp. Immunol..

[39] Sitja-Bobadilla A., Palenzuela O., Woo P.T.K., Buchmann K. (2012). *Enteromyxum* species. Fish Parasites: Pathobiology and Protection.

[40] Frazer L.N. (2009). Sea-cage aquaculture, sea lice, and declines of wild fish. Conserv. Biol..

[41] Moller O.S., Woo P.T.K., Buchmann K. (2012). *Argulus* *foliaceus*. Fish Parasites: Pathobiology and Protection.

[42] Bartholomew J.L., Reno P.W. (2002). The history and dissemination of whirling disease. Am. Fish. Soc. Symp..

[43] Sudhagar A., Kumar G., El-Matbouli M. (2020). The malacosporean myxozoan parasite *Tetracapsuloides bryosalmonae*: A threat to wild salmonids. Pathogens.

[44] Hallett S.L., Ray R.A., Hurst C.N., Holt R.A., Buckles G.R., Atkinson S.D., Bartholomew J.L. (2012). Density of the waterborne parasite *Ceratomyxa shasta* and its biological effects on salmon. Appl. Environ. Microbiol..

[45] Dickerson H.W., Findly R.C. (2014). Immunity to *Ichthyophthirius* infections in fish: A synopsis. Dev. Comp. Immunol..

[46] Barker S.E., Bricknell I.R., Covello J., Purcell S., Fast M.D., Wolters W., Bouchard D.A. (2019). Sea lice, *Lepeophtheirus salmonis* (Krøyer 1837), infected Atlantic salmon (*Salmo salar* L.) are more susceptible to infectious salmon anemia virus. PLoS ONE.

[47] Smith N.C., Rise M.L., Christian S.L. (2019). A Comparison of the Innate and Adaptive Immune Systems in Cartilaginous Fish, Ray-Finned Fish, and Lobe-Finned Fish. Front. Immunol..

[48] Rauta P.R., Nayak B., Das S. (2012). Immune system and immune responses in fish and their role in comparative immunity study: A model for higher organisms. Immunol. Lett..

[49] Picard-Sánchez A., Estensoro I., del Pozo R., Piazzon M.C., Palenzuela O., Sitjà-Bobadilla A. (2019). Acquired protective immune response in a fish-myxozoan model encompasses specific antibodies and inflammation resolution. Fish Shellfish Immunol..

[50] Rigos G., Pavlidis M., Divanach P. (2001). Host susceptibility to Cryptocaryon sp. infection of Mediterranean marine broodfish held under intensive culture conditions: A case report. Bull. Eur. Assoc. Fish. Pathol..

[51] Takano T., Kondo H., Hirono I., Endo M., Saito-Taki T., Aoki T. (2011). Toll-like receptors in teleosts. Diseases in Asian Aquaculture VII.

[52] Sudhagar A., El-Matbouli M., Kumar G. (2020). Identification and expression profiling of toll-like receptors of brown trout (*Salmo trutta*) during proliferative kidney disease. Int. J. Mol. Sci..

[53] Zhao F., Li Y.W., Pan H.J., Shi C.B., Luo X.C., Li A.X., Wu S.Q. (2013). Expression profiles of toll-like receptors in channel catfish (*Ictalurus punctatus*) after infection with *Ichthyophthirius multifiliis*. Fish Shellfish Immunol..

[54] Li Y.W., Xu D.D., Li X., Mo Z.Q., Luo X.C., Li A.X., Dan X.M. (2016). Identification and characterization of three TLR1 subfamily members from the orange-spotted grouper, *Epinephelus coioides*. Dev. Comp. Immunol..

[55] Valenzuela-Muñoz V., Boltaña S., Gallardo-Escárate C. (2016). Comparative immunity of *Salmo salar* and *Oncorhynchus kisutch* during infestation with the sea louse *Caligus rogercresseyi*: An enrichment transcriptome analysis. Fish Shellfish Immunol..

[56] Becker I., Salaiza N., Aguirre M., Delgado J., Carrillo-Carrasco N., Kobeh L.G., Ruiz A., Cervantes R., Torres A.P., Cabrera N. (2003). *Leishmania* lipophosphoglycan (LPG) activates NK cells through toll-like receptor. Mol. Biochem. Parasitol..

[57] Sepulcre M., Pelegrín P., Mulero V., Meseguer J. (2002). Characterisation of gilthead seabream acidophilic granulocytes by a monoclonal antibody unequivocally points to their involvement in fish phagocytic response. Cell Tissue Res..

[58] Palikova M., Papezikova I., Markova Z., Navratil S., Mares J., Mares L., Vojtek L., Hyrsl P., Jelinkova E., Schmidt-Posthaus H. (2017). Proliferative kidney disease in rainbow trout (*Oncorhynchus mykiss*) under intensive breeding conditions: Pathogenesis and haematological and immune parameters. Vet. Parasitol..

[59] Muñoz P., Álvarez-Pellitero P., Sitja-Bobadilla A. (2000). Modulation of the in vitro activity of European sea bass (*Dicentrarchus labrax* L.) phagocytes by the myxosporean parasite *Sphaerospora dicentrarchi* (Myxosporea: Bivalvulida). Fish Shellfish Immunol..

[60] Chin A., Woo P.T.K. (2005). Innate cell-mediated immune response and peripheral leukocyte populations in Atlantic salmon, *Salmo salar* L.; to a live *Cryptobia salmositica* vaccine. Parasitol. Res..

[61] Korytář T., Wiegertjes G.F., Zusková E., Tomanová A., Lisnerová M., Patra S., Sieranski V., Šíma R., Born-Torrijos A., Wentzel A.S. (2019). The kinetics of cellular and humoral immune responses of common carp to presporogonic development of the myxozoan *Sphaerospora molnari*. Parasites Vectors.

[62] Graves S.S., Evans D.L., Dawe D.L. (1985). Mobilization and activation of nonspecific cytotoxic cells (ncc) in the channel catfish (*Ictalurus punctatus*) infected with *Ichthyophthirius multifiliis*. Comp. Immunol. Microbiol. Infect. Dis..

[63] Mali P., Sanyal K.B., Mukherjee D., Guchhait A., Dash G. (2017). Nonspecific cytotoxic cells (NCC) in fish: A review. J. Interacad..

[64] Jaso-Friedmann L., Leary J.H., Weisman Z., Evans D.L. (1996). Activation of nonspecific cytotoxic cells with a multiple antigenic peptide: Specificity and requirements for receptor crosslinkage. Cell. Immunol..

[65] Leiro J., Piazzón M.C., Budiño B., Sanmartín M.L., Lamas J. (2008). Complement-mediated killing of *Philasterides dicentrarchi* (Ciliophora) by turbot serum: Relative importance of alternative and classical pathways. Parasite Immunol..

[66] Agius C., Roberts R.J. (2003). Melano-macrophage centres and their role in fish pathology. J. Fish Dis..

[67] Cuesta A., Muñoz P., Rodríguez A., Salinas I., Sitjà-Bobadilla A., Álvarez-Pellitero P., Esteban M.A., Meseguer J. (2006). Gilthead seabream (*Sparus aurata* L.) innate defence against the parasite *Enteromyxum leei* (Myxozoa). Parasitology.

[68] Holzer A.S., Schachner O. (2001). Myxosporidia and macrophage centres in chub (*Leuciscus cephalus*)-Quantitative interactions focus on *Myxobolus cyprini*. Parasitology.

[69] Sitjà-Bobadilla A., Redondo M.J., Bermúdez R., Palenzuela O., Ferreiro I., Riaza A., Quiroga I., Nieto J.M., Alvarez-Pellitero P. (2006). Innate and adaptive immune responses of turbot, *Scophthalmus maximus* (L.), following experimental infection with *Enteromyxum scophthalmi* (Myxosporea: Myxozoa). Fish Shellfish Immunol..

[70] Davey G.C., Calduch-Giner J.A., Houeix B., Talbot A., Sitjà-Bobadilla A., Prunet P., Pérez-Sánchez J., Cairns M.T. (2011). Molecular profiling of the gilthead sea bream (*Sparus aurata* L.) response to chronic exposure to the myxosporean parasite *Enteromyxum leei*. Mol. Immunol..

[71] Baerwald M.R. (2013). Temporal expression patterns of rainbow trout immune-related genes in response to *Myxobolus cerebralis* exposure. Fish Shellfish Immunol..

[72] Bjork S.J., Zhang Y.A., Hurst C.N., Alonso-Naveiro M.E., Alexander J.D., Sunyer J.O., Bartholomew J.L. (2014). Defenses of susceptible and resistant Chinook salmon (*Onchorhynchus tshawytscha*) against the myxozoan parasite *Ceratomyxa shasta*. Fish Shellfish Immunol..

[73] Wang B., Wangkahart E., Secombes C.J., Wang T. (2019). Insights into the evolution of the suppressors of cytokine signaling (SOCS) gene family in vertebrates. Mol. Biol. Evol..

[74] Kotob M.H., Kumar G., Saleh M., Gorgoglione B., Abdelzaher M., El-Matbouli M. (2018). Differential modulation of host immune genes in the kidney and cranium of the rainbow trout (*Oncorhynchus mykiss*) in response to *Tetracapsuloides bryosalmonae* and *Myxobolus cerebralis* co-infections. Parasites Vectors.

[75] Jørgensen L.v.G. (2017). The fish parasite *Ichthyophthirius multifiliis*—Host immunology, vaccines and novel treatments. Fish Shellfish Immunol..

[76] Mustafa A., MacWilliams C., Fernandez N., Matchett K., Conboy G.A., Burka J.F. (2000). Effects of sea lice (*Lepeophtheirus salmonis* Kröyer, 1837) infestation on macrophage functions in Atlantic salmon (*Salmo salar* L.). Fish Shellfish Immunol..

[77] Dalvin S., Jørgensen L.v.G., Kania P.W., Grotmol S., Buchmann K., Øvergård A.C. (2020). Rainbow trout *Oncorhynchus mykiss* skin responses to salmon louse *Lepeophtheirus salmonis*: From copepodid to adult stage. Fish Shellfish Immunol..

[78] Wallace J.L. (2005). Nitric oxide as a regulator of inflammatory processes. Mem. Inst. Oswaldo Cruz.

[79] Salinas I., Zhang Y.A., Sunyer J.O. (2011). Mucosal immunoglobulins and B cells of teleost fish. Dev. Comp. Immunol..

[80] Saulnier D., De Kinkelin P. (1996). Antigenic and biochemical study of PKX, the myxosporean causative agent of proliferative kidney disease of salmonid fish. Dis. Aquat. Organ..

[81] Kumar G., Abd-Elfattah A., El-Matbouli M. (2014). Differential modulation of host genes in the kidney of brown trout *Salmo trutta* during sporogenesis of *Tetracapsuloides bryosalmonae* (Myxozoa). Vet. Res..

[82] Hedrick R.P., El-Matbouli M., Adkison M.A., MacConnell E. (1998). Whirling disease: Re-emergence among wild trout. Immunol. Rev..

[83] Muñoz P., Palenzuela O., Sitjà-Bobadilla A., Álvarez-Pellitero P. (1999). Immunohistochemical reactivity of polyclonal antibodies against *Sphaerospora testicularis* and *Ceratomyxa labracis* (Myxosporea: Bivalvulida), with other myxosporean parasites. Int. J. Parasitol..

[84] Bartholomew J.L. (1998). Host resistance to infection by the myxosporean parasite *Ceratomyxa shasta*: A review. J. Aquat. Anim. Health.

[85] Sitjà-Bobadilla A., Redondo M.J., Macias M.A., Ferreiro I., Riaza A., Alvarez-Pellitero P. (2004). Development of immunohistochemistry and enzyme-linked immunosorbent assays for the detection of circulating antibodies against *Enteromyxum scophthalmi* (Myxozoa) in turbot (*Scophthalmus maximus* L.). Fish Shellfish Immunol..

[86] Zhao Y., Liu X., Sato H., Zhang Q., Li A., Zhang J. (2019). RNA-seq analysis of local tissue of *Carassius auratus* gibelio with pharyngeal myxobolosis: Insights into the pharyngeal mucosal immune response in a fish-parasite dialogue. Fish Shellfish Immunol..

[87] Zhang Y.A., Salinas I., Li J., Parra D., Bjork S., Xu Z., Lapatra S.E., Bartholomew J., Sunyer J.O. (2010). IgT, a primitive immunoglobulin class specialized in mucosal immunity. Nat. Immunol..

[88] Xu Z., Parra D., Gómez D., Salinas I., Zhang Y.A., Von Gersdorff Jørgensen L., Heinecke R.D., Buchmann K., LaPatra S., Oriol Sunyer J. (2013). Teleost skin, an ancient mucosal surface that elicits gut-like immune responses. Proc. Natl. Acad. Sci. USA.

[89] Abos B., Estensoro I., Perdiguero P., Faber M., Hu Y., Rosales P.D., Granja A.G., Secombes C.J., Holland J.W., Tafalla C. (2018). Dysregulation of B cell activity during proliferative kidney disease in rainbow trout. Front. Immunol..

[90] MacKinnon B.M. (1993). Host Response of Atlantic Salmon (*Salmo salar*) to Infection by Sea Lice (*Caligus elongatus*). Can. J. Fish. Aquat. Sci..

[91] Nakanishi T., Shibasaki Y., Matsuura Y. (2015). T cells in fish. Biology.

[92] Saleh M., Montero R., Kumar G., Sudhagar A., Friedl A., Köllner B., El-Matbouli M. (2019). Kinetics of local and systemic immune cell responses in whirling disease infection and resistance in rainbow trout. Parasites Vectors.

[93] Tarleton R.L. (2005). New approaches in vaccine development for parasitic infections. Cell. Microbiol..

[94] Clem A.S. (2011). Fundamentals of vaccine immunology. J. Glob. Infect. Dis..

[95] Goven B.A., Dawe D.L., Gratzek J.B. (1980). Protection of channel catfish, *Ictalurus punctatus* Rafmesque, against *Ichthyophthirius multifiliis* Fouquet by immunization. J. Fish. Biol..

[96] Gibbons I.R. (1963). Studies on the Protein Components of Cilia From *Tetrahymena pyriformis*. Proc. Natl. Acad. Sci. USA.

[97] Sleigh M.A. (1974). Cilia and Flagella.

[98] Burkart M.A., Clark T.G., Dickerson H.W. (1990). Immunization of channel catfish, *Ictalurus punctatus* Rafinesque, against *Ichthyophthirius multifiliis* (Fouquet): Killed versus live vaccines. J. Fish Dis..

[99] Xu D.H., Zhang D., Shoemaker C., Beck B. (2019). Immune response of channel catfish (*Ictalurus punctatus*) against *Ichthyophthirius multifiliis* post vaccination using DNA vaccines encoding immobilization antigens. Fish Shellfish Immunol..

[100] Xu D.H., Klesius P.H. (2013). Comparison of serum antibody responses and host protection against parasite *Ichthyophthirius multifiliis* between channel catfish and channel × blue hybrid catfish. Fish Shellfish Immunol..

[101] Von Gersdorff Jørgensen L., Kania P.W., Rasmussen K.J., Mattsson A.H., Schmidt J., Al-Jubury A., Sander A., Salanti A., Buchmann K. (2017). Rainbow trout (*Oncorhynchus mykiss*) immune response towards a recombinant vaccine targeting the parasitic ciliate *Ichthyophthirius multifiliis*. J. Fish. Dis..

[102] von Gersdorff Jørgensen L., Sigh J., Kania P.W., Holten-Andersen L., Buchmann K., Clark T., Rasmussen J.S., Einer-Jensen K., Lorenzen N. (2012). Approaches towards DNA Vaccination against a Skin Ciliate Parasite in Fish. PLoS ONE.

[103] Jørgensen L.V.G., Nemli E., Heinecke R.D., Raida M.K., Buchmann K. (2008). Immune-relevant genes expressed in rainbow trout following immunisation with a live vaccine against *Ichthyophthirius multifiliis*. Dis. Aquat. Organ..

[104] Dalgaard M., Buchmann K., Li A. (2002). Immunization of rainbow trout fry with *Ichthyophthirius multifiliis* sonicate: Protection of host and immunological changes. Bull. Eur. Assoc. Fish Pathol..

[105] Xu D.H., Klesius P.H., Shoemaker C.A. (2008). Protective immunity of Nile tilapia against *Ichthyophthirius multifiliis* post-immunization with live theronts and sonicated trophonts. Fish Shellfish Immunol..

[106] Yambot A.V., Song Y.L. (2006). Immunization of grouper, *Epinephelus coioides*, confers protection against a protozoan parasite, *Cryptocaryon irritans*. Aquaculture.

[107] Grayson T.H., John R.J., Wadsworth S., Greaves K., Cox D., Roper J., Wrathmell A.B., Gilpin M.L., Harris J.E. (1995). Immunization of Atlantic salmon against the salmon louse: Identification of antigens and effects on louse fecundity. J. Fish Biol..

[108] Faber M.N., Holland J.W., Secombes C.J. (2019). Vaccination strategies and IgM responses against PKD in rainbow trout. Fish Shellfish Immunol..

[109] Kane S.N., Mishra A., Dutta A.K. (2016). Preface: International Conference on Recent Trends in Physics (ICRTP 2016). J. Phys. Conf. Ser..

[110] Harikrishnan R., Balasundaram C., Heo M.S. (2012). Poly d,l-lactide-co-glycolic acid (PLGA)-encapsulated vaccine on immune system in *Epinephelus bruneus* against *Uronema marinum*. Exp. Parasitol..

[111] Fontenla F., Blanco-Abad V., Pardo B.G., Folgueira I., Noia M., Gómez-Tato A., Martínez P., Leiro J.M., Lamas J. (2016). Vaccine-induced modulation of gene expression in turbot peritoneal cells. A microarray approach. Mol. Immunol..

[112] Méndez S., Gurunathan S., Kamhawi S., Belkaid Y., Moga M.A., Skeiky Y.A.W., Campos-Neto A., Reed S., Seder R.A., Sacks D. (2001). The Potency and Durability of DNA- and Protein-Based Vaccines Against *Leishmania major* Evaluated Using Low-Dose, Intradermal Challenge. J. Immunol..

[113] Knox D.P., Redmond D.L. (2006). Parasite vaccines-Recent progress and problems associated with their development. Parasitology.

[114] Mutoloki S., Munang’andu H.M., Evensen Ø. (2015). Oral vaccination of fish-antigen preparations, uptake, and immune induction. Front. Immunol..

[115] Bøgwald J., Dalmo R.A. (2019). Review on immersion vaccines for fish: An update. Microorganisms.

[116] Plant K.P., LaPatra S.E. (2011). Advances in fish vaccine delivery. Dev. Comp. Immunol..

[117] Horne M.T. (1997). Technical aspects of the administration of vaccines. Dev. Biol. Stand..

[118] Sitjà-Bobadilla A., Estensoro I., Pérez-Sánchez J. (2016). Immunity to gastrointestinal microparasites of fish. Dev. Comp. Immunol..

[119] Woo P.T.K. (2003). *Cryptobia* (*Trypanoplasma*) *salmositica* and salmonid cryptobiosis. J. Fish. Dis..

[120] Sitjà-Bobadilla A., Palenzuela O., Alvarez-Pellitero P. (2008). Immune response of turbot, *Psetta maxima* (L.) (Pisces: Teleostei), to formalin-killed scuticociliates (Ciliophora) and adjuvanted formulations. Fish Shellfish Immunol..

[121] Buchmann K., Lindenstrøm T., Bresciani J. (2001). Defence mechanisms against parasites in fish and the prospect for vaccines. Acta Parasitol.

[122] Katzenback B.A., Plouffe D.A., Haddad G., Belosevic M. (2008). Administration of recombinant parasite β-tubulin to goldfish (*Carassius auratus* L.) confers partial protection against challenge infection with *Trypanosoma danilewskyi* Laveran and Mesnil. Vet. Parasitol..

[123] Buchmann K. (2020). Immune response to *Ichthyophthirius multifiliis* and role of IgT. Parasite Immunol..

[124] Mo Z.Q., Li Y.W., Wang H.Q., Wang J.L., Ni L.Y., Yang M., Lao G.F., Luo X.C., Li A.X., Dan X.M. (2016). Comparative transcriptional profile of the fish parasite *Cryptocaryon irritans*. Parasites Vectors.

[125] Piazzon M.C., Wiegertjes G.F., Leiro J., Lamas J. (2011). Turbot resistance to *Philasterides dicentrarchi* is more dependent on humoral than on cellular immune responses. Fish Shellfish Immunol..

[126] Griffiths K.L., Khader S.A. (2014). Novel vaccine approaches for protection against intracellular pathogens. Curr. Opin. Immunol..

[127] Bethony J., Loukas A., Smout M., Brooker S., Mendez S., Plieskatt J., Goud G., Bottazzi M.E., Zhan B., Wang Y. (2005). Antibodies against a secreted protein from hookworm larvae reduce the intensity of hookworm infection in humans and vaccinated laboratory animals. FASEB J..

[128] Riveau G., Deplanque D., Remoué F., Schacht A.M., Vodougnon H., Capron M., Thiry M., Martial J., Libersa C., Capron A. (2012). Safety and immunogenicity of rSh28GST antigen in humans: Phase 1 randomized clinical study of a vaccine candidate against urinary schistosomiasis. PLoS Negl. Trop. Dis..

[129] Lofthouse S.A., Andrews A.E., Elhay M.J., Bowles V.M., Meeusen E.N.T., Nash A.D. (1996). Cytokines as adjuvants for ruminant vaccines. Int. J. Parasitol..

[130] Brown G.V., Reeder J.C. (2002). Malaria vaccines. Med. J. Aust..

[131] Arkoosh M.R., Kaattari S.L. (1991). Development of immunological memory in rainbow trout (*Oncorhynchus mykiss*). I. An immunochemical and cellular analysis of the B cell response. Dev. Comp. Immunol..

[132] Ferguson H.W., BalL H.J. (1979). Epidemiological aspects of proliferative kidney disease amongst rainbow trout *Salmo gairdneri* Richardson in Northern Ireland. J. Fish Dis..

[133] Findly R.C., Zhao X., Noe J., Camus A.C., Dickerson H.W. (2013). B cell memory following infection and challenge of channel catfish with *Ichthyophthirius multifiliis*. Dev. Comp. Immunol..

[134] Gorgoglione B., Wang T., Secombes C.J., Holland J.W. (2013). Immune gene expression profiling of Proliferative Kidney Disease in rainbow trout *Oncorhynchus mykiss* reveals a dominance of anti-inflammatory, antibody and T helper cell-like activities. Vet. Res..

[135] Fast M.D. (2014). Fish immune responses to parasitic copepod (namely sea lice) infection. Dev. Comp. Immunol..

[136] Dzikowski R., Deitsch K. (2006). Antigenic variation by protozoan parasites: Insights from *Babesia bovis*. Mol. Microbiol..

[137] El-Matbouli M., Hoffmann R.W., Schoel H., McDowell T.S., Hedrick R.P. (1999). Whirling disease: Host specificity and interaction between the actinosporean stage of *Myxobolus cerebralis* and rainbow trout *Oncorhynchus mykiss*. Dis. Aquat. Organ..

[138] Clark T.G., Mcgraw R.A., Dickerson H.W. (1992). Developmental expression of surface antigen genes in the parasitic ciliate *Ichthyophthirius multifiliis*. Proc. Natl. Acad. Sci. USA.

[139] Palti Y. (2011). Toll-like receptors in bony fish: From genomics to function. Dev. Comp. Immunol..

[140] Nóbrega de Sousa T., de Menezes Neto A., Alves de Brito C.F. (2013). “Omics” in the study of the major parasitic diseases malaria and schistosomiasis. Infect. Genet. Evol..

[141] Amambua-Ngwa A., Tetteh K.K.A., Manske M., Gomez-Escobar N., Stewart L.B., Deerhake M.E., Cheeseman I.H., Newbold C.I., Holder A.A., Knuepfer E. (2012). Population Genomic Scan for Candidate Signatures of Balancing Selection to Guide Antigen Characterization in Malaria Parasites. PLoS Genet..

[142] Hull R., Dlamini Z. (2014). The role played by alternative splicing in antigenic variability in human endo-parasites. Parasites Vectors.

[143] Yeoh L.M., Goodman C.D., Mollard V., McHugh E., Lee V.V., Sturm A., Cozijnsen A., McFadden G.I., Ralph S.A. (2019). Alternative splicing is required for stage differentiation in malaria parasites. Genome Biol..

[144] Haase S., Cabrera A., Langer C., Treeck M., Struck N., Herrmann S., Jansen P.W., Bruchhaus I., Bachmann A., Dias S. (2008). Characterization of a conserved rhoptry-associated leucine zipper-like protein in the malaria parasite *Plasmodium falciparum*. Infect. Immun..

[145] Lindner S.E., Swearingen K.E., Harupa A., Vaughan A.M., Sinnis P., Moritz R.L., Kappe S.H.I. (2013). Total and putative surface proteomics of malaria parasite salivary gland sporozoites. Mol. Cell. Proteom..

[146] Doolan D.L. (2011). Plasmodium Immunomics. Int. J. Parasitol.

[147] Doolan B.D.L., Sedegah M., Hedstrom R.C., Charoenvit P.H., Hoffrnan S.L. (1996). Circumventing Genetic Restriction of Protection against Malaria with Multigene DNA Immunization: CD8 + T Cell, Interferon, and Nitric Oxide-Dependent Immunity. J. Exp. Med..

[148] Osier F.H.A., Fegan G., Polley S.D., Murungi L., Verra F., Tetteh K.K.A., Lowe B., Mwangi T., Bull P.C., Thomas A.W. (2008). Breadth and magnitude of antibody responses to multiple *Plasmodium falciparum* merozoite antigens are associated with protection from clinical malaria. Infect. Immun..

[149] Cecílio P., Pérez-Cabezas B., Fernández L., Moreno J., Carrillo E., Requena J.M., Fichera E., Reed S.G., Coler R.N., Kamhawi S. (2017). Pre-clinical antigenicity studies of an innovative multivalent vaccine for human visceral leishmaniasis. PLoS Negl. Trop. Dis..

[150] Jayaraj R., Piedrafita D., Dynon K., Grams R., Spithill T.W., Smooker P.M. (2009). Vaccination against fasciolosis by a multivalent vaccine of stage-specific antigens. Vet. Parasitol..

[151] Kumar G., Kocour M. (2017). Applications of next-generation sequencing in fisheries research: A review. Fish. Res..

[152] Sudhagar A., Ertl R., Kumar G., El-Matbouli M. (2019). Transcriptome profiling of posterior kidney of brown trout, *Salmo trutta*, during proliferative kidney disease. Parasites Vectors.

[153] Kumar G., Ertl R., Bartholomew J.L., El-Matbouli M. (2020). First transcriptome analysis of bryozoan *Fredericella sultana*, the primary host of myxozoan parasite *Tetracapsuloides bryosalmonae*. PeerJ.

[154] Kumar G., Ertl R., Bartholomew J.L., El-Matbouli M. (2020). Transcriptome analysis elucidates the key responses of bryozoan *Fredericella sultana* during the development of *Tetracapsuloides bryosalmonae* (Myxozoa). Int. J. Mol. Sci..

[155] Kumar G., Ertl R., Nilsen F., Bartholomew J.L., El-Matbouli M. (2021). Data of de novo transcriptome assembly of the myxozoan parasite *Tetracapsuloides bryosalmonae*. Data Br..

[156] Ronza P., Robledo D., Bermúdez R., Losada A.P., Pardo B.G., Sitjà-Bobadilla A., Quiroga M.I., Martínez P. (2016). RNA-seq analysis of early enteromyxosis in turbot (*Scophthalmus maximus*): New insights into parasite invasion and immune evasion strategies. Int. J. Parasitol..

[157] Hartigan A., Kosakyan A., Pecková H., Eszterbauer E., Holzer A.S. (2020). Transcriptome of *Sphaerospora molnari* (Cnidaria, Myxosporea) blood stages provides proteolytic arsenal as potential therapeutic targets against sphaerosporosis in common carp. BMC Genom..

[158] Chang E.S., Neuhof M., Rubinstein N.D., Diamant A., Philippe H., Huchon D., Cartwright P. (2015). Genomic insights into the evolutionary origin of Myxozoa within Cnidaria. Proc. Natl. Acad. Sci. USA.

[159] Mo Z.Q., Yang M., Wang H.Q., Xu Y., Huang M.Z., Lao G.F., Li Y.W., Li A.X., Luo X.C., Dan X.M. (2016). Grouper (*Epinephelus coioides*) BCR signaling pathway was involved in response against *Cryptocaryon irritans* infection. Fish Shellfish Immunol..

[160] Kumar G., Gotesman M., El-Matbouli M. (2015). Interaction of *Tetracapsuloides bryosalmonae*, the causative agent of proliferative kidney disease, with host proteins in the kidney of *Salmo trutta*. Parasitol. Res..

[161] Piriatinskiy G., Atkinson S.D., Park S., Morgenstern D., Brekhman V., Yossifon G., Bartholomew J.L., Lotan T. (2017). Functional and proteomic analysis of *Ceratonova shasta* (Cnidaria: Myxozoa) polar capsules reveals adaptations to parasitism. Sci. Rep..

[162] Saleh M., Kumar G., Abdel-Baki A.A.S., Dkhil M.A., El-Matbouli M., Al-Quraishy S. (2019). Quantitative proteomic profiling of immune responses to *Ichthyophthirius multifiliis* in common carp skin mucus. Fish Shellfish Immunol..

[163] Mai Y.Z., Li Y.W., Li R.J., Li W., Huang X.Z., Mo Z.Q., Li A.X. (2015). Proteomic analysis of differentially expressed proteins in the marine fish parasitic ciliate *Cryptocaryon irritans*. Vet. Parasitol..

[164] Coyne R.S., Hannick L., Shanmugam D., Hostetler J.B., Brami D., Joardar V.S., Johnson J., Radune D., Singh I., Badger J.H. (2011). Comparative genomics of the pathogenic ciliate *Ichthyophthirius multifiliis*, its free-living relatives and a host species provide insights into adoption of a parasitic lifestyle and prospects for disease control. Genome Biol..

[165] Chen K., Wang J.L., Huang S.Y., Yang W.B., Zhu W.N., Zhu X.Q. (2017). Immune responses and protection after DNA vaccination against *Toxoplasma gondii* calcium-dependent protein kinase 2 (TgCDPK2). Parasite.

[166] Zhao G., Zhou A., Lu G., Meng M., Sun M., Bai Y., Han Y., Wang L., Zhou H., Cong H. (2013). Identification and characterization of *Toxoplasma gondii* aspartic protease 1 as a novel vaccine candidate against toxoplasmosis. Parasites Vectors.

[167] Overington J.P., Al-Lazikani B., Hopkins A.L. (2006). How many drug targets are there?. Nat. Rev. Drug Discov..

[168] National Centre for Biotechnology Information. https://www.ncbi.nlm.nih.gov/bioproject/PRJNA241036/.

[169] National Centre for Biotechnology Information. https://www.ncbi.nlm.nih.gov/sra/SRX4615721/.

[170] Hahn C., Fromm B., Bachmann L. (2014). Comparative genomics of flatworms (Platyhelminthes) reveals shared genomic features of ecto-and endoparastic neodermata. Genome Biol. Evol..

[171] National Centre for Biotechnology Information. https://www.ncbi.nlm.nih.gov/sra/?term=SRX2417163.

[172] National Centre for Biotechnology Information. https://www.ncbi.nlm.nih.gov/sra/?term=SRX2417144.

[173] Ivory C., Chadee K. (2004). DNA vaccines: Designing strategies against parasitic infections. Genet. Vaccines Ther..

[174] Budiño B., Lamas J., Pata M.P., Arranz J.A., Sanmartín M.L., Leiro J. (2011). Intraspecific variability in several isolates of *Philasterides dicentrarchi* (syn. *Miamiensis avidus*), a scuticociliate parasite of farmed turbot. Vet. Parasitol..

