# Supplementary figures and images for: Development of Fish Parasite Vaccines in the OMICs Era: Progress and Opportunities
Source: Vaccines (Basel). 2021 Feb 20;9(2):179. doi: 10.3390/vaccines9020179 (PMC7923790; doi:10.3390/vaccines9020179)

**Figure S1:** PRISMA flow diagram of study search and selection

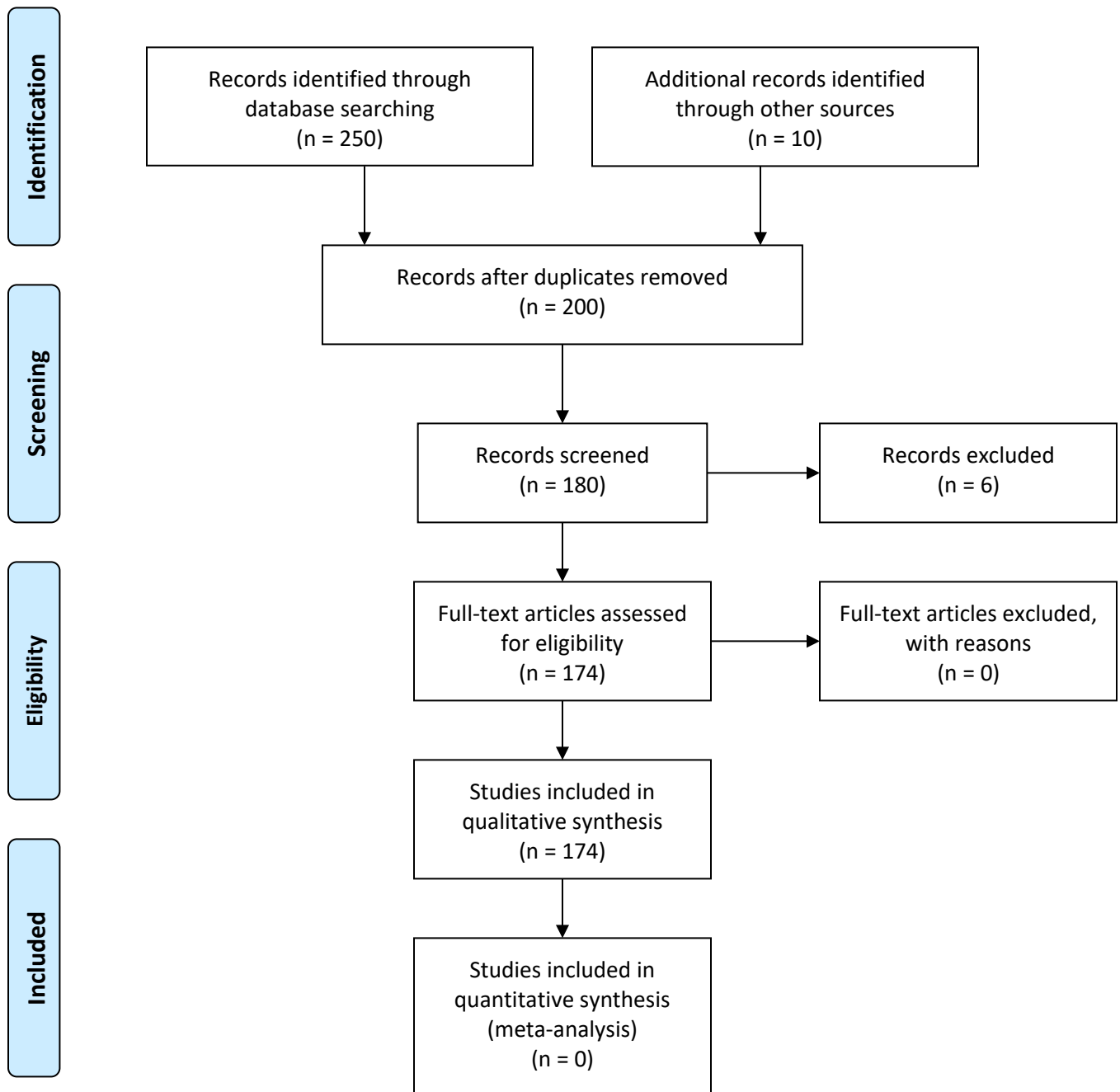

Supplement: Supplementary file 1 [file vaccines-09-00179-s001.pdf]
